# Double primary malignancies associated with colon cancer in patients with situs inversus totalis: two case reports

**DOI:** 10.1186/1477-7819-9-109

**Published:** 2011-09-23

**Authors:** Young Wan Kim, Hoon Ryu, Dae Sung Kim, Ik Yong Kim

**Affiliations:** 1 Department of Surgery, Yonsei University Wonju Health System, 162 Ilsan-dong, Wonju-si, Gangwon-do, (220-701), Korea

**Keywords:** Double primary cancer, Situs inversus totalis, Gastrointestinal malignancy

## Abstract

Situs inversus totalis (SIT) is not itself a premalignant condition, however, rare synchronous or metachronous multiple primary malignancies have been reported. Herein we present a case of synchronous transverse and sigmoid colon cancers and a case of metachronous rectosigmoid colon and gastric cancers in patients with SIT.

A 66-year-old male with SIT was referred for a two-month history of hematochezia. Synchronous colonic tumors were found on the proximal transverse and sigmoid colon. The patient underwent open total colectomy and was discharged without incident. A 71-year-old female with rectosigmoid colon cancer and SIT underwent laparoscopy-assisted low anterior resection. Fourteen months after the surgery, the patient developed a single hepatic metastasis and underwent hepatic segmentectomy (S6). Forty-six months after laparoscopy-assisted low anterior resection, the patient developed metachronous early gastric cancer on the antrum and underwent radical subtotal gastrectomy with gastroduodenostomy. The patient is doing well without recurrence for 28 months.

## Background

The anatomical arrangement of human organs is described by three categories: situs solitus, situs inversus, and situs ambiguus. Situs solitus indicates the normal arrangement of the heart and intra-abdominal organs. Situs inversus is a rare congenital deformity and is classified as either situs inversus totalis (SIT) or partial situs inversus. Unlike partial situs inversus, in cases of SIT, the left and right aspects of the thoracic and intra-abdominal organs are inverted like a mirror image [1]. Situs ambiguus refers to disorderly arranged thoracic and abdominal organs unlike the features of situs solitus and situs inversus [2]. The incidence rate of SIT is 1 per 10,000-20,000 people [3] and, in a recent Korean study, 5 per 34,723 SIT patients were discovered during colonoscopy [4].

Situs inversus itself is not a premalignant condition; however, rare synchronous and metachronous multiple primary gastrointestinal malignancies have been reported in the literature. On review of the Korean literature, one case of synchronous gastric and esophageal cancers in a 49-year-old male with SIT [5] and one case of synchronous esophageal and gastric cancers in a 58-year-old male with situs ambiguus with polysplenia were reported [6]. Herein, we present a case of synchronous transverse and sigmoid colon cancer and a case of metachronous rectosigmoid colon and gastric cancer in SIT patients. To our knowledge, this paper is the first report of double primary malignancies associated with colon cancer in patients with SIT in Korea.

## Case presentation

### (Case 1)

**Figure 1:**
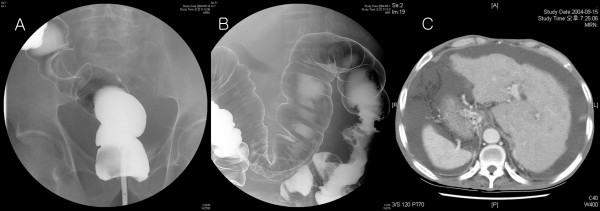
**Synchronous double primary cancer in a 66-year-old male patient with situs inversus totalis**.**a** Barium double contrast study showing an ulcerative tumor in the sigmoid colon. **b** Barium double contrast study showing an ulcerative tumor in the proximal transverse colon. **c** An abdominal computed tomography scan shows liver cirrhosis with splenomegaly and a large amount of ascites.

A 66-year-old male was referred to the Colorectal Surgical Department for a two-month history of hematochezia. The patient had been diagnosed with alcoholic liver cirrhosis four years prior. Barium double contrast study and colonoscopy showed ulcerative tumors on the proximal transverse and sigmoid colon (Figures 1a and 1b). An abdominal computed tomography (CT) scan showed liver cirrhosis with splenomegaly and a large amount of ascites (Figure 1c). Echocardiography and chest x-ray showed dextrocardia and the abdominal CT scan showed complete inversion of the internal organs. Laboratory investigation showed a hemoglobin level of 9.1 g/dL, and an albumin level of 2.8 g/dL. Other blood tests were unremarkable. The patient underwent an open total colectomy with ileo-rectal anastomosis and was discharged uneventfully on postoperative day 18. Pathologic results showed serosal invasive (T3) adenocarcinomas in the proximal transverse and sigmoid colon. Lymph node metastasis was found in 4 out of 23 nodes. The patient died of cirrhosis-related liver failure three months after the surgery.

### (Case 2)

**Figure 2:**
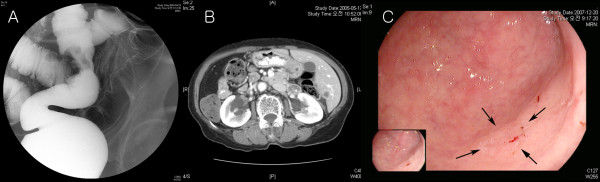
**Metachronous double primary cancer in 71-year-old female patient with situs inversus totalis**. **a** Barium double contrast study showing an ulcerative tumor in the rectosigmoid colon. **b** An abdominal computed tomography scan showing a single 9-mm metastatic nodule in the liver (segment 6) 14 months after laparoscopy-assisted low anterior resection. **c** Upper gastrointestinal endoscopy shows an ill-defined 5-mm mucosal lesion on the antrum 46 months after laparoscopy-assisted low anterior resection (black arrows).

A 71-year-old female was referred to the Colorectal Surgical Department for a four-month history of tenesmus and intermittent hematochezia. Physical examination and routine laboratory studies were unremarkable. Barium double contrast study and colonoscopy showed ulcerofungating tumors on the rectosigmoid colon (Figure 2a). An abdominal computed tomography (CT) scan showed no hepatic metastasis. Echocardiography and chest x-ray showed dextrocardia and the abdominal CT scan showed complete inversion of the abdominal organs. The patient underwent laparoscopy-assisted low anterior resection. There were no postoperative complications and the patient was discharged on postoperative day 15. Pathologic results showed that adenocarcinoma infiltrated the pericolic fat (T3) with no lymph node metastasis (0/13). Thereafter, the patient received adjuvant chemotherapy using a 5-fluorouracil and leucovorin regimen for six months. Fourteen months after laparoscopy-assisted low anterior resection, the patient developed a single hepatic metastasis and underwent hepatic segmentectomy (S6) (Figure 2b). Forty-six months after laparoscopy-assisted low anterior resection, the patient developed metachronous early gastric cancer on the antrum and underwent radical subtotal gastrectomy with gastroduodenostomy (Figure 2c). Pathologic results showed that gastric adenocarcinoma was confined to the mucosa (T1) and there was no lymph node metastasis. The patient is doing well without recurrence 28 months after gastrectomy.

In the general population, the reported incidence of synchronous colon cancer ranges from 3 to 8% [7], and the incidence of metachronous colon cancer ranges from 0.5 to 3.6% [8]. According to Galiatsatos et al. [9], 41 cases of malignancy in patients with situs abnormalities including SIT and situs ambiguus were found in the English literature from 1980 to 2005, in addition to three cases of multiple primary cancer. The incidence of multiple primary cancer (3/41, 7.3%) did not differ between patients with situs solitus and patients with situs abnormalities [10].

The occurrence of malignancy in patients with situs abnormalities may be sporadic, though some have suggested a possible relationship between situs abnormalities and cancer. One theory is that unidentified genes affecting left-right axis arrangement may be related to cancer susceptibility. The pathogenesis underlying this hypothesis has not been well characterized [9].

The diagnosis of synchronous gastrointestinal malignancy or metachronous malignancy during follow-up is straightforward due to advances in endoscopy and radiological imaging. However, before surgical treatment, careful preoperative anatomic evaluation is important because situs abnormalities may accompany major vascular anomalies [11]. Fortunately, we did not encounter any vascular anomalies in our cases.

In this study, when performing laparoscopic surgery in patients with rectosigmoid colon cancer, the operator was positioned on the left side of the patient, which is opposite the position used for a normal patient. The right-handed operating surgeon had difficulties performing distal rectal transection through a left lower quadrant port and thus used four endoscopic staple cartridges. Oms and Badia [12] proposed that a left-handed surgeon may have a technical advantage during laparoscopy in patients with SIT; however Jobanputra et al. [13] indicated that right-handedness did not preclude performing laparoscopy [14].

## Conclusions

Although double primary gastrointestinal malignancies associated with colon cancer in patients with SIT are very rare, the possibility of synchronous lesions or the occurrence of metachronous cancer of the alimentary tract should not be overlooked, and careful preoperative evaluation is important for successful surgical treatment.

## Consent

Written informed consent was obtained from the patient for publication of this Case report and any accompanying images. A copy of the written consent is available for review by the Editor-in-Chief of this journal.

## List of abbreviations

SIT: Situs inversus totalis.

## Competing interests

The authors declare that they have no competing interests.

## Authors' contributions

IYK took part in the design. HR and DSK acquired the data. YWK drafted the manuscript and made revisions. All authors read and approved the final manuscript.
